# BMI-stratified outcomes of a badminton training program on health-related fitness in adults with mild to moderate intellectual disabilities

**DOI:** 10.3389/fpsyt.2025.1625301

**Published:** 2025-11-06

**Authors:** Lechen Zhu, Xiaohuan Tan, Tao Li, Yifan Wang

**Affiliations:** 1 School of Physical Education, Shanghai University of Sport, Shanghai, China; 2 School of Exercise and Health, Shanghai University of Sport, Shanghai, China

**Keywords:** badminton exercise, physical fitness, BMI, intellectual disabilities, adult

## Abstract

**Background:**

Regular exercise, such as that obtained through badminton, can effectively improve health issues associated with insufficient physical activity among adults with intellectual disabilities (ID). However, there is a paucity of research exploring tailored interventions and post-exercise outcomes among adults with ID based on body mass index (BMI) stratifications. This study compared the effects of a 12-week badminton intervention on health-related physical fitness in adults with ID across three BMI levels, providing a theoretical reference for developing targeted exercise prescriptions for this population.

**Methods:**

In total, 60 adults (39 male and 21 female) 25–45 years of age with mild to moderate ID were included and allocated to one of three analysis groups based on their BMI (20 per group): normal weight (BMI: 18.5–24.99 kg/m²), overweight (BMI: 25.0–29.9 kg/m²), and obesity (BMI ≥30.0 kg/m²). A systematic 12-week badminton training intervention was conducted, with two 65-minute sessions per week. Health-related physical fitness assessments were performed before and after the intervention. Key outcome measures included (1) aerobic capacity (assessed using the 2-minute step test, resting heart rate, and vital capacity), (2) muscle strength and endurance (assessed through grip strength, standing long jump, sit-ups, and the 30-second chair stand test), and (3) flexibility and coordination (assessed via the sit and reach test, back scratch test, and timed up-and-go test). Data were analyzed using SPSS 22.0 for within-group and between-group comparisons.

**Results:**

Baseline health-related physical fitness indicators showed no significant differences among the normal weight, overweight, and obesity groups pre-intervention (p > 0.05). Completion of the badminton exercise program resulted in differential improvements. Significant enhancements were observed for all three groups in aerobic capacity (2-minute step test, resting heart rate, and vital capacity), muscle strength and endurance (right-hand grip strength, standing long jump, and 30-second chair stand test), and coordination (timed up-and-go test), after undergoing pre-and post-tests (p < 0.05). Multivariate analysis of variance (MANOVA) indicating differences between groups showed that compared with the overweight group, the normal weight group had greater improvements in right-hand grip strength; compared with the obesity group, the normal weight group exhibited greater improvements observed in right-hand grip strength and performance in the timed up-and-go test; and compared with the obesity group, the overweight group demonstrated more pronounced reductions in resting heart rate and better timed up-and-go test performance (p < 0.05). There was no significant change in flexibility (sit and reach test, back scratch test) between pre-test and post-test performances. MANOVA results for intergroup analyses showed no significant improvements in the 2-minute step test, lung capacity, left grip strength, sit-ups, standing long jump, sit and reach test, and back scratch test.

**Conclusion:**

Badminton exercise significantly improved aerobic capacity, muscle strength and endurance and coordination in adults with ID across all BMI categories. Core fitness indicators (right-hand grip strength, timed up-and-go test, and resting heart rate) exhibited a gradient improvement pattern: normal weight greater improvement than overweight, which was greater than obesity. Therefore, although badminton is an appropriate exercise intervention for adults with ID, BMI and BMI-related factors should be taken into consideration when designing personalized exercise programs to optimize training effects.

## 1 Introduction

Intellectual disability (ID) is a highly heterogeneous neurodevelopmental disorder characterized by significant limitations in intellectual functioning and adaptive behavior (1, 2), typically occurring before 18 years of age (3). Based on the severity of functional impairment, ID is classified into four levels: mild, moderate, severe, and profound (see **Table 1**) (4).

**Table 1:** Grading of intellectual disability(^4^).

Grade	Intellectual developmental level		Social adaptive functioning	
Level	Developmental quotient Age 0-6 years	Intelligence quotient Age ≥7 years	Adaptive behavior	WHO-DAS II value Ages ≥18 years
1	≤25	≤20	Profound	≥116
2	26-39	20-34	Severe	106-115
3	40-54	35-49	Moderate	96-105
4	55-75	50-69	Mild	52-95

WHO-DAS II, World Health Organization Disability Assessment Scale II.

Compared with children with ID, adults with ID may achieve some degree of skill independence but face greater challenges in workplaces or communities, with their quality of life and independent living abilities heavily reliant on societal support (5). Due to a lack of positive awareness about the benefits of health-related physical fitness (6), adults with ID often exhibit low motivation to engage in exercise, leading to physical inactivity, poor motor skills, and a tendency toward sedentary lifestyles (7). This inactivity increases risks of secondary consequences, such as osteoarthritis, declines in balance, strength, endurance, and coordination (8, 9), and higher rates of chronic diseases and all-cause mortality (10), ultimately compromising their quality of life (11).

Physical exercise is recognized as a critical and widely utilized intervention to improve physical health and social adaptation for individuals with ID (12). Regular exercise effectively prevents health issues associated with insufficient physical activity in this population (8), and previous studies have demonstrated the efficacy of badminton in enhancing health outcomes for individuals with ID (12). Badminton, a net-based racquet sport that involves hitting a shuttlecock with a stringed racket, combines physical exertion with recreational appeal (13). Given the cognitive and communication limitations of individuals with ID (14), they often struggle to engage in complex sports or to articulate health needs. Badminton movements have relatively simple techniques, strong repeatability, a controllable environment, and low risk of injury. They do not require high cognitive and language communication skills, making them more easily accepted and mastered by people with ID. Importantly, badminton, as one of the most popular sports worldwide, is highly accepted and participated in by the adult community (15, 16). Badminton provides opportunities for interactions with others, which helps to enhance the confidence of individuals with ID and reduce social isolation (17). This makes badminton an ideal intervention tool for adults with ID.

Although numerous studies have investigated the impact of physical activity interventions in people with ID, most studies have focused on evaluating exercise interventions in obesity subgroups, leaving a significant gap in understanding differential intervention effects across weight categories, particularly regarding body mass index (BMI) stratification (normal, overweight, and obesity) and associated exercise benefits. Different BMI levels may affect the ability to execute some exercise-related skills among people with ID, as weight levels may affect exercise efficiency, endurance, and overall performance (18). For example, individuals with obesity may face a greater physical burden during exercise, resulting in poor performance and excessive joint load, whereas among people of normal weight and overweight, lower physical exertion may lead to better endurance and require higher exercise goals. The impact of BMI is not limited to the athletic performance itself; it may also indirectly affect participation in exercise through its direct effects on physical health. For example, people with ID having heavier body weight may be more prone to fatigue, shortness of breath, or discomfort, thereby reducing their willingness and motivation to participate in exercise. In contrast, people with ID having normal weight may participate in exercise more easily, achieving better physical adaptation and health improvement effects. Therefore, different levels of BMI may have a moderating or reinforcing effect on the effectiveness of exercise interventions, thereby affecting exercise abilities and health benefits among people with ID (19). However, there remains a paucity of research exploring tailored interventions and post-exercise outcomes for distinct BMI groups among adults with ID. Thus, establishing a precision-based exercise prescription system aligned with these BMI classifications holds critical value (8).

Therefore, this study compared the effects of badminton exercise on indices of health-related physical fitness across BMI categories (normal weight, overweight, and obesity groups) among adults with ID to identify optimal intervention outcomes.

## 2 Participants and methods

### 2.1 Study participants

We recruited adults with mild to moderate ID from Sunshine Base, a non-profit aid organization that provides vocational rehabilitation and skills training for people with disabilities. The criteria for study inclusion were (1) having an IQ score range of 50–69 that was certified by a mental health center; (2) holding a disability certificate; (3) having no prior badminton training experience; and (4) having an absence of physical disabilities or chronic medical conditions.

### 2.2 Experimental methods

This study was approved by the Ethics Committee of Shanghai University of Sport (approval No. 102772021RT067). Participants voluntarily agreed to participate in the study and understood that they had the right to terminate or withdraw from the study at any time. Written informed consent was obtained from the participant, a responsible person at the Sunshine Base, and the participant’s parents or guardians prior to the start of the study.

**Figure 1:**
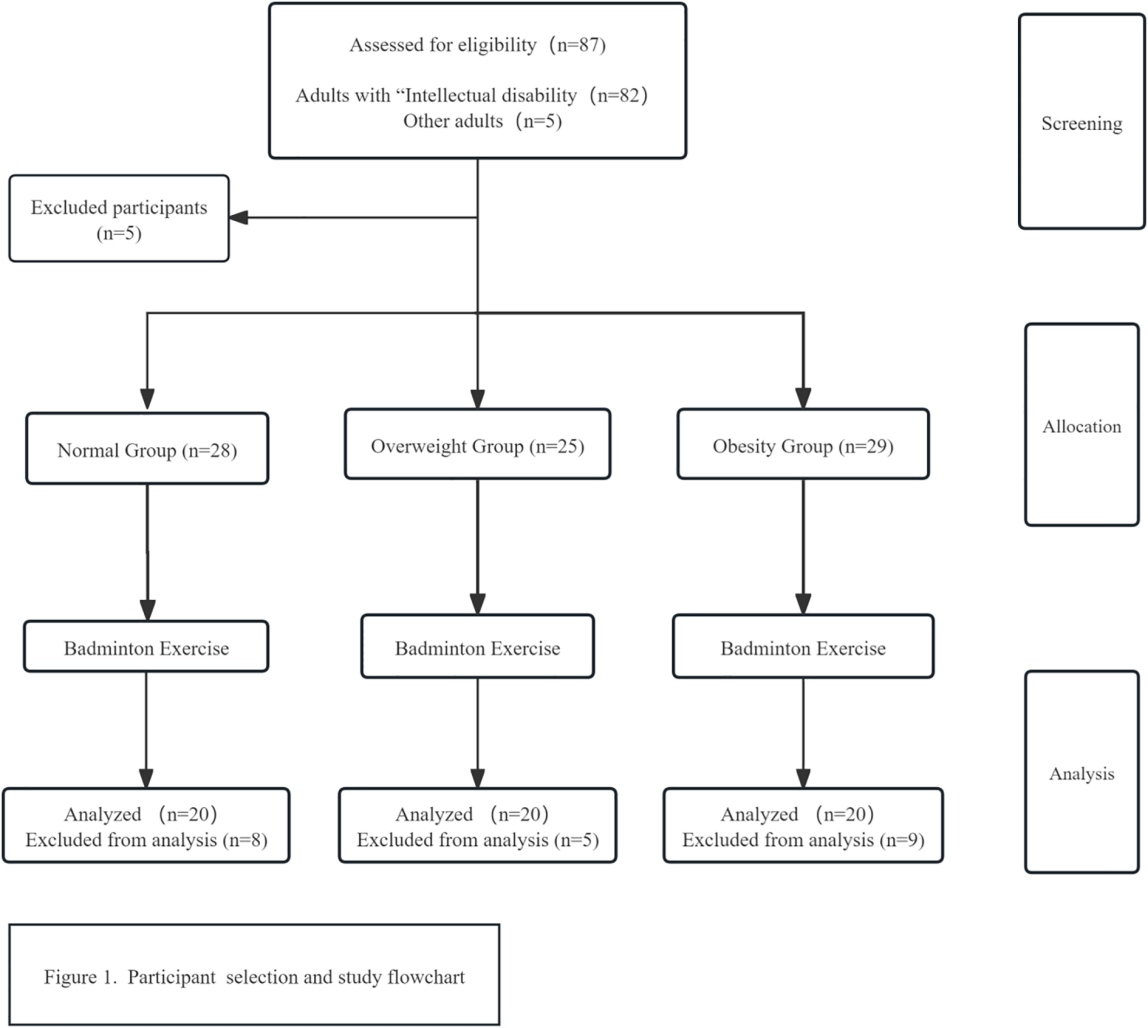
Participant selection and study flowchart.

This study recruited 82 adults with ID who met the criteria. A total of 22 people withdrew or were absent from one-third of the intervention due to health or personal reasons and were, therefore, excluded from the analysis. In total, 60 adults 25–45 years of age with mild to moderate ID were included in the experimental analysis (39 male and 21 female; 55 participants right-hand dominant, and 5 left-hand dominant). In order to reduce measurement bias in the experiment, the testers were unaware of group allocations during the experiment. Prior to group allocation, participants were grouped based on observational information from their pre-test BMI to prevent them from being randomly assigned to BMI groups during the intervention. **Figure 1** shows the details for participant screening, group allocation, and analysis through the study, and the participants’ basic characteristics are shown in **Table 2**. Eligible participants were assigned to one of three groups based on their BMI category: Normal (BMI: 18.5–24.99 kg/m²), Overweight (BMI: 25.0–29.9 kg/m²), and Obesity (BMI ≥30.0 kg/m²) (20).

**Table 2:** Exercise plan.

Study week	Practice or drill	Pre- & post-session activities	Total no. of repetitions
1	Forehand grip and swing practice (15 min) Forehand multiball practice (25 min)	Stationary body weight circuit (10 min) Dynamic stretching and cool-down (5 min)	3
2	Backhand grip and swing practice (15 min) Backhand multiball practice (25 min)	Stationary body weight circuit (10 min) Dynamic stretching and cool down (5 min)	3
3	Basic footwork drills in badminton (15 min) Forehand multiball practice (25 min)	Stationary body weight circuit (10 min) Dynamic stretching and cool down (5 min)	3
4	Basic footwork drills in badminton (15 min) Backhand multiball practice (25 min)	Stationary body weight circuit (10 min) Dynamic stretching and cool down (5 min)	3
5	Forehand net lift practice (20 min) Backhand net lift practice (20 min)	Stationary body weight circuit (10 min) Dynamic stretching and cool down (5 min)	3
6	Forehand serve practice (20 min) Backhand serve practice (20 min)	Stationary body weight circuit (10 min) Dynamic stretching and cool down (5 min)	3
7-8	Clear shot swing practice (10 min) Clear shot swing practice (30 min)	Stationary body weight circuit (10 min) Dynamic stretching and cool down (5 min)	6
9	Footwork while moving practice (15 min) Forehand net shot technique (25 min)	Stationary body weight circuit (10 min) Dynamic stretching and cool down (5 min)	6
10-11	Explanation of badminton rules (20 min) Net shot practice in badminton (20 min)	Stationary body weight circuit (10 min) Dynamic stretching and cool down (5 min)	6
12	Badminton friendly match across the net (40 min)	Stationary body weight circuit (10 min) Dynamic stretching and cool down (5 min)	6

A 12-week badminton training program was conducted with two sessions per week, and data analysis was performed to evaluate the impact of badminton training on the health and physical fitness of adults with mild to moderate ID. The exercise plan that participants followed is shown in **Table 3**.

**Table 3:** Participant characteristics.

Group	No.	Age, M ± SD (years)	Height, M ± SD (m)	Body weight, M ± SD (kg)	BMI M ± SD (kg/m²)
Normal	20	40.1±7.65	1.67±0.12	61.99±11.39	22.175±2.09
Overweight	20	38.3±6.51	1.71±0.11	75.9±10.43	26.27±0.97
Obesity	20	39.1±7.52	1.65±0.11	88.95±13.65	32.73±3.20

### 2.3 Experimental design

This study used a 3 × 2 mixed experimental design (between-group factor × time factor), with a between-group factor of weight category (as defined above) and a time factor (within-subject factor) that comprised baseline pre-test and post-test assessment periods. The experimental process was divided into three stages: (1) the baseline assessment phase, in which all participants underwent standardized pre-testing, followed by (2) a 12-week structured badminton intervention and (3) multidimensional post-testing completed within 1 week after the intervention. All testing and health assessments took place in the activity room of the Sunshine Base. Each participant’s testing session lasted approximately 20 minutes, with appropriate rest intervals between tests.

### 2.4 Exercise program

The initial exercise program phase focused on structured racket swing training as the core intervention. A breakdown teaching method was used to establish standard racket swing movements, with an emphasis on optimizing the coordination and force generation mechanism of the shoulder, elbow, and wrist joints. In the early stages, multiball targeted training was used to develop hand-eye coordination, gradually progressing to dynamic hitting. Dual-task training (hitting + counting) was implemented to enhance cognitive–motor integration.

Each study session consisted of three stages. In the preparation phase (15 min), participants jogged and performed dynamic stretching. The main phase (40 min) consisted of ball practice. The ending phase (10 min) was reserved for stretching again. All phases used positive behavior support strategies and specific praise techniques (e.g., “Today, the number of your successful shots to the target area increased by 20%.”) to enhance self-efficacy.

Training implementation followed the progressive principle, that is, the training content gradually progressed from simple to difficult. A dynamic evaluation mechanism was set up, with 5%-10% intensity adjustments based on weekly assessment results to ensure that the training load was within the zone of proximal development. Safety was maintained during training by physiological monitoring, with intermittent heart rate (HR) monitoring performed every 10 minutes when the duration of the badminton exercise exceeded 30 minutes. In addition, before, during, and after each session, the integrity of the racket handle and the tension of the net were checked. Furthermore, during the training process, assistance was provided to help individuals with ID learn the correct movements and to promptly correct any incorrect actions to avoid injury caused by prolonged incorrect movements. The badminton program details are shown in **Table 3**.

### 2.5 Measurements

#### 2.5.1 Aerobic capacity

##### 2.5.1.1 Two-minute step test

Cardiopulmonary endurance was measured using a 2-minute step test. Before the test, the participant stood upright and was instructed to maintain a stable stepping frequency while performing continuous alternating steps with the thigh raised to a level that was parallel to the ground. At the start command, the participant began stepping in place. The number of steps completed within 2 minutes with the right knee reaching the required height was recorded (21).

##### 2.5.1.2 Heart rate

The proxy for cardiovascular health was resting HR. During the measurement of resting HR, the surrounding environment was quiet. The participant rested in a seated position for at least 5 minutes before the measurement and was instructed to avoid tension and to relax their muscles. HR was measured on the right upper arm using an HR monitor. The data were manually transferred to a computer (22).

##### 2.5.1.3 Vital capacity

Aerobic endurance was measured through vital capacity. The participants were instructed to inhale deeply and then exhale as forcefully as possible without pausing during the exhalation into a disinfected spirometer mouthpiece inserted into a spirometer tube. The maximum value from two tests was recorded (23).

#### 2.5.2 Muscular strength and endurance

##### 2.5.2.1 Grip strength

Upper body muscle strength was measured using a dynamometer. The participant maintained an upright posture and was instructed to grip the dynamometer handle in one hand as firmly as possible. The data on the dynamometer screen changed until no new peak measurements were recorded (24). After practicing twice, the formal test began. Participants were allowed two test attempts, and the best result from each hand was recorded.

##### 2.5.2.2 Standing long jump

Lower body muscle strength was measured using the standing long jump. Participants stood with their feet shoulder width apart and toes parallel and behind a line that was drawn on clean, flat ground. The participant jumped from there, without any stepping or continuous jumping movements ahead of the jump. The distance from the line to the heel of the foot nearest the line when landing was recorded. Each participant performed two tests, and the best result was recorded (25).This method has shown good feasibility and test-retest reliability (intraclass correlation coefficient range, 0.90-0.99) (26).

##### 2.5.2.3 30-second sit-ups

Abdominal muscle endurance was measured using sit-ups. Participants lay on a yoga mat with their knees bent and feet flat on the ground. Their lower legs formed a 90°-angle with the thighs, and both hands were placed beside the ears. The tester placed their hands on the participant’s ankles to provide support. At the tester’s command, the participants used their abdominal muscles to lift the upper body, with the arms reaching toward the knees. The participant then returned to the starting position to complete one repetition. The total number of sit-ups completed by the participant within 30 seconds was recorded (27).

##### 2.5.2.4 30-second chair stand test

Lower body muscle endurance was measured using the sit-to-stand test. Participants sat in a chair with their feet flat on the floor, shoulder width apart, and with the soles of their feet completely in contact with the ground. The back remained upright, and the arms were crossed over the chest. At the tester’s command, the participant stood fully and then sat to the starting position to complete one repetition. The total number of repetitions completed by the participant within 30 seconds was recorded (28).

#### 2.5.3 Flexibility and coordination

##### 2.5.3.1 Sit and reach test

Flexibility of the upper and lower limbs was measured using the sit and reach test, which primarily assesses the range of motion of the torso, waist, hips, and other joints at rest. During the test, participants sat with their back against a wall, legs straight, heels together, and toes apart. Both arms were extended forward, with hands placed on the instrument board. The participants slowly bent the upper body forward, pushing the slider on the instrument until they could no longer move forward, at which point the test ended. Participants were allowed to practice twice before two formal test attempts. The best result of the two tests was recorded.

##### 2.5.3.2 Back scratch test

Upper limb flexibility was measured using the back scratch test. The participant was instructed to reach one hand downward over the shoulder and the other hand upward behind the back, attempting to overlap the hands as much as possible. A shorter distance between the hands indicated better upper back and shoulder flexibility. During the test, participants were required to keep the back upright and avoid bending forward. They held the position briefly after completing the movement to assess whether the hands could successfully overlap. A ruler was used to measure the distance (in centimeters) between the middle fingers of both hands. A negative score indicated the distance between non-overlapping fingers (e.g., -5 cm), whereas a positive score indicated the amount of overlap (e.g., +3 cm). The test was performed twice, and the best score was retained as the final result.

##### 2.5.3.3 Timed up-and-go test

Lower limb coordination and gait control were measured using the TUG test. The participants sat on a chair with feet flat on the floor and knees parallel to the ground. Both hands were placed at their sides or on their knees. To start the test, the participant stood, walked 5 meters as quickly as possible without stopping, turned around, and returned to the starting position, sitting down to complete the test (29). Timing started when the participant’s buttocks left the chair, and stopped when the participant returned to the chair and sat completely, with buttocks touching the chair surface.

### 2.6 Statistical analysis

Statistical analysis was performed using Microsoft Excel and SPSS, version 22.0 software. The health and fitness indicators of the three groups of adults with ID before the badminton intervention were tested using the Kolmogorov-Smirnov test. All indicators in the groups followed a normal distribution. Descriptive statistics are presented as mean ± standard deviation (M ± SD). Paired sample t-tests were used to compare the pre- and post-test result differences within the same group. A multivariate analysis of variance was used to compare the differences between groups before and after the intervention. The least significant difference (LSD) test is appropriate when multivariate analysis of variance (MANOVA) results indicate significant differences and the number of experimental groups is small (e.g., 3 groups). In this case, the additional increase in type I error risk from using LSD is minimal. Thus, LSD can capture true differences without compromising the interpretation of the results. By contrast, the Bonferroni method for multiple comparisons may be too conservative, leading to some true differences being missed. Because the LSD method does not substantially adjust the significance level, it improves the sensitivity of the test (30). Therefore, pairwise comparisons were conducted using the LSD test. A p-value less than 0.05 was considered statistically significant.

## 3 Results

Comparisons of all health and fitness indicators evaluated in this study before and after the badminton intervention for adults with mild to moderate ID across different BMI levels are shown in **Table 4**.

**Table 4:** Differences in health-related physical fitness metrics before vs after the badminton intervention in normal, overweight, and obesity groups.

Test	Normal group		Overweight group		Obesity group		Normal group P value	Overweight group P value	Obesity group P value
	Pre-intervention	Post-intervention	Pre-intervention	Post-intervention	Pre-intervention	Post-intervention			
2- minute Step Test (steps)	107.05±29.19	119.55±28.35	111.23±15.33	122.59±20.63	103.10±21.07	122.85±29.95	0.020*	0.004**	0.002**
HR (bpm)	92.90±17.31	85.00±15.75	88.71±11.93	81.05±9.56	94.95±12.39	88.20±9.62	0.010**	0.004**	0.022*
Vital Capacity (mL)	1816.60±90.58	2179.80±997.77	2122.00±987.71	2300.20±902.87	2105.50±1208.64	2369.75±1173.32	0.021*	0.024*	0.014*
Grip Strength, Left (kg)	22.58±8.04	24.05±9.92	26.91±9.42	27.80±8.53	25.07±11.41	26.50±10.82	0.157	0.333	0.169
Grip Strength, Right (kg)	22.68±8.38	26.64±8.45	26.34±9.45	29.93±7.46	27.73±9.30	29.80±10.32	0.001**	0.019*	0.016*
Standing Long Jump (cm)	111.45±47.60	127.45±49.17	116.90±37.85	129.60±31.53	97.95±43.22	116.80±33.99	0.001**	0.015*	0.003**
Sit-Ups (30 seconds, repetitions)	9.38±5.00	10.10±4.44	11.20±3.90	12.35±2.58	7.75±4.56	8.25±5.07	0.327	0.083	0.344
30-Second Chair Stand Test (repetitions)	15.74±4.38	13.47±3.24	16.63±2.97	14.05±2.74	15.80±4.69	13.05±3.38	0.012*	0.001**	0.003**
Sit and Reach Test (cm)	-2.31±7.49	-1.48±6.20	-5.01±6.68	-4.09±6.96	-8.25±9.19	-7.14±6.01	0.249	0.370	0.454
Back Scratch Test (cm)	-3.79±7.10	-1.42±8.57	-6.55±16.30	-3.13±9.97	-7.95±7.27	-6.15±6.84	0.061	0.100	0.182
TUG (seconds)	9.01±2.13	10±1.85	8.05±1.71	7.52±1.63	10.31±2.85	9.30±2.40	0.006**	0.001**	0.029*

*P < 0.05; **P ≤ 0.01

### 3.1 Aerobic capacity indicators

**Figure 2:**
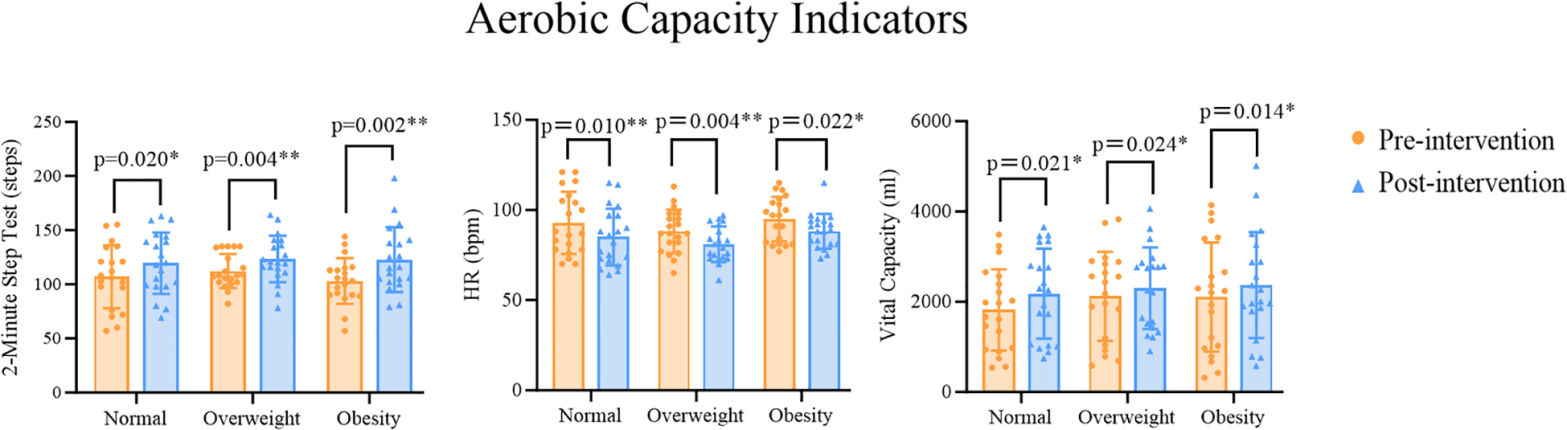
*p<0.05 indicates statistically significant differences, **p<0.01 indicates differences with greater statistical significance.

Paired sample t-tests were used to analyze changes in aerobic capacity indicators across the three groups of participants. Compared with the pre-test within the same group, the post-test performance showed significant differences for the 2-minute step test in the Normal (t=-2.548, p=0.020), Overweight (t=-3.230, p=0.004), and Obesity (t=-3.559, p=0.002) Groups. For HR, there were also significant differences in the Normal (t=2.848, p=0.010), Overweight (t=3.204, p=0.004), and Obesity (t=2.484, p=0.022) Groups between the pre- and post-test performances. Similarly for vital capacity, significant differences were found in the Normal (t=-2.506, p=0.021), Overweight (t=-2.452, p=0.024), and Obesity (t=-2.707, p=0.014) Groups between the pre- and post-test performances (**Figure 2**).

Multivariate analysis of variance (MANOVA) results showed that there were significant differences in some aerobic capacity indicators between different BMI groups when comparing pre- and post-test performances. Further *post-hoc* comparisons (LSD) revealed no significant differences in the changed performances for the 2-minute step test between the groups (Normal vs Overweight: p=0.417; Normal vs Obesity: p=0.953; Overweight vs Obesity: p=0.385). The Obesity Group had lower pre- and post-test performances on the 2-minute step test than the Normal Group, and the Normal Group had lower performances on the 2-minute step test than the Overweight Group. For HR as an indicator of cardiovascular health, there were no significant differences in the changed performances between the Normal Group and the Overweight Group or the Obesity Group (Normal vs Overweight: p=0.143; Normal vs Obesity: p=0.373), but there was a significant difference in the changed performances between the Overweight and Obesity Groups (p=0.020). The Overweight Group had lower pre- and post-test performances on resting HR than the Normal Group, and the Normal Group had lower performances on resting HR than the Obesity Group, with the Obesity Group significantly higher than the Overweight Group. For the vital capacity test, there were no significant differences in the changed performances between the groups (Normal vs Overweight: p=0.360; Normal vs Obesity: p=0.301; Overweight vs Obesity: p=0.909). The Normal Group had lower pre- and post-test performances on the vital capacity test than the Overweight Group, and the Overweight Group had lower performances on the vital capacity test than the Obesity Group (**Table 5**).

**Table 5:** Post hoc multiple comparisons of aerobic capacity among normal, overweight, and obesity groups.

Indicator test	Group A	Group B	Mean group A	Mean group B	Difference (A-B)	P value
	Normal	Overweight	113.30	117.83	-4.53	0.417
2- minute Step Test (steps)	Overweight	Obesity	117.83	112.98	4.85	0.385
	Obesity	Normal	112.98	113.30	-0.33	0.953
	Normal	Overweight	88.95	84.63	4.33	0.143
HR (bpm)	Overweight	Obesity	84.63	91.58	-6.95	0.020*
	Obesity	Normal	91.58	88.95	2.63	0.373
	Normal	Overweight	1998.20	2211.10	-212.90	0.360
Vital Capacity (mL)	Overweight	Obesity	2211.10	2237.63	-26.53	0.909
	Obesity	Normal	2237.63	1998.20	239.43	0.301

*p<0.05.

### 3.2 Muscle strength and endurance indicators

**Figure 3:**
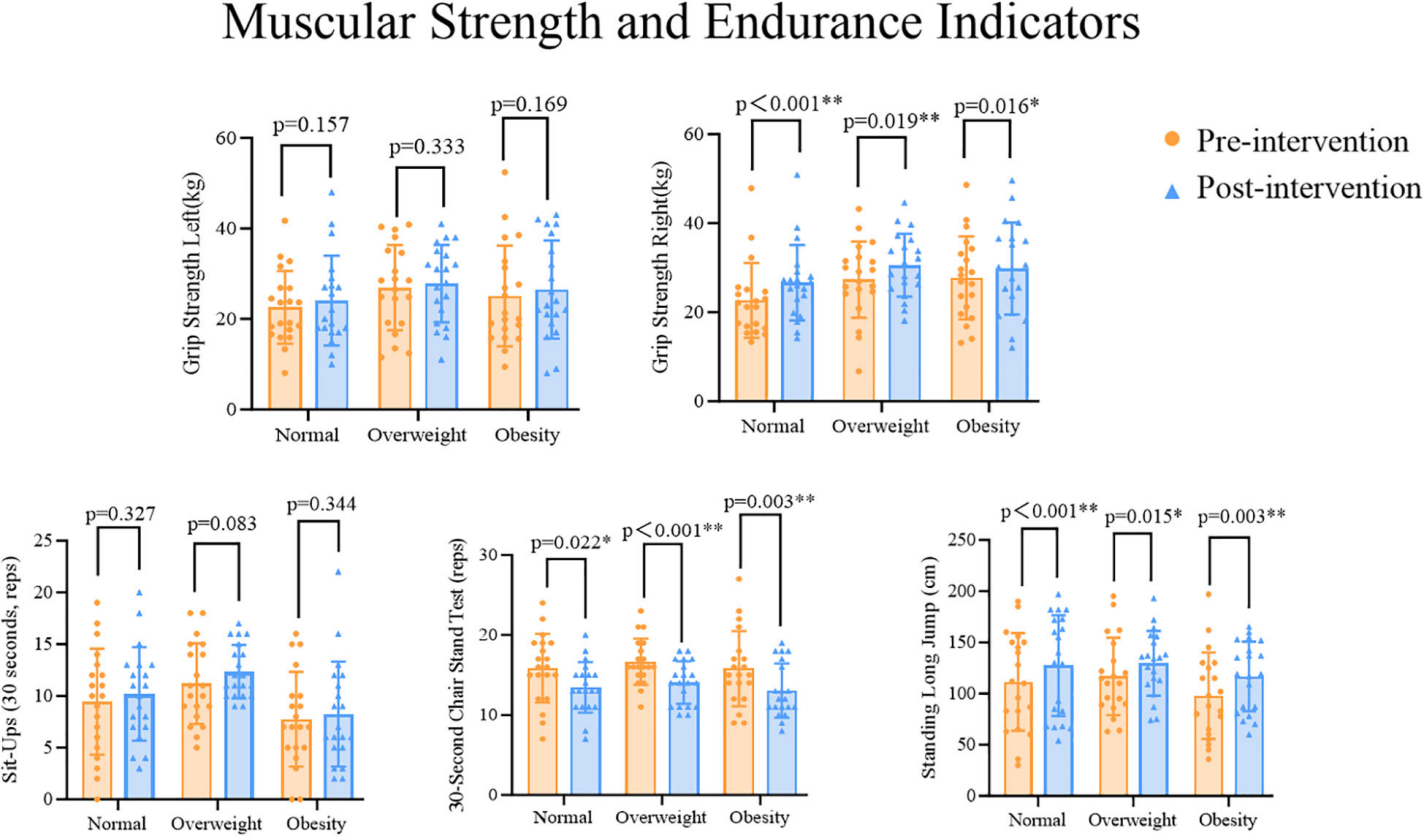
*p<0.05 indicates statistically significant differences, **p<0.01 indicates differences with greater statistical significance.

Paired sample t-tests were used to analyze changes in muscle strength and endurance indicators across the three groups of participants. The results showed that compared with the pre-test performances within the same group, there were no significant differences in the post-test performances for left-hand grip strength in the Normal (t=-1.472, p=0.157), Overweight (t=-0.993, p=0.333), or Obesity (t=-1.431, p=0.169) Groups. By contrast, for right-hand grip strength, significant differences were found between the pre- and post-test performances for the Normal (t=-3.983, p=0.001), Overweight (t=-2.560, p=0.019), and Obesity (t=-2.641, p=0.016) Groups. For the standing long jump, significant differences were also found in the post-test performances compared with the pre-test performances for the Normal (t=-3.976, p=0.001), Overweight (t=-2.678, p=0.015), and Obesity (t=-3.368, p=0.003) Groups. For the sit-up indicator, there were no significant differences in the post-test performances for the Normal (t=-1.005, p=0.327), Overweight (t=-1.827, p=0.083), and Obesity (t=-0.970, p=0.344) Groups. For the sit-to-stand indicator, significant differences were found in the post-test performances for the Normal (t=-3.983, p=0.012), Overweight (t=3.939, p=0.001), and Obesity (t=3.477, p=0.003) Groups (**Figure 3**).

MANOVA results showed that there were significant differences in some muscle strength and endurance indicators between different BMI groups when comparing pre- and post-test performances. Further *post-hoc* comparisons (LSD) revealed the following results. For left-hand grip strength, there were no significant differences in the changed performances between the groups (Normal vs Overweight: p=0.065; Normal vs Obesity: p=0.257; Overweight vs Obesity: p=0.471). The Normal Group had lower performances on left-hand grip strength than the Obesity Group, and the Obesity Group had lower performances on left-hand grip strength than the Overweight Group. However, for right-hand grip strength, there were significant differences in the changed performances between the Normal and Overweight Groups, as well as between the Normal and Obesity Groups (Normal vs Overweight: p=0.031; Normal vs Obesity: p=0.038), but no significant difference was found between the Overweight and Obesity Groups (Overweight vs Obesity: p=0.933). The Normal Group had lower performances on right-hand grip strength than the Obesity Group, and the Obesity Group had lower performances on right-hand grip strength than the Overweight Group, with the Normal Group performances on right-hand grip strength significantly lower than both the Overweight and Obesity Group performances. For the standing long jump indicator, there were no significant differences in the changed performances between the groups (Normal vs Overweight: p=0.679; Normal vs Obesity: p=0.190; Overweight vs Obesity: p=0.085). The Obesity Group had lower performances on the standing long jump indicator than both the Normal and Overweight Groups. For the sit-up indicator, there were significant differences in the changed performances between the Normal and Overweight Groups, as well as between the Overweight and Obesity Groups (Normal vs Overweight: p=0.049; Overweight vs Obesity: p=0.001). However, no significant difference was found between the Normal and Obesity Groups (Normal vs Obesity: p=0.065). The Normal Group had significantly higher performances on the sit-up indicator than the Overweight Group and the Overweight Group had higher performances on the this indicator than the Obesity Group, while the Obesity Group had the lowest performances on the sit-up indicator. For the sit-to-stand indicator, there were no significant differences in the changed performances between the groups (Normal vs Overweight: p=0.385; Normal vs Obesity: p=0.78; Overweight vs Obesity: p=0.251). The Obesity Group had lower performances on the sit-to-stand indicator than both the Normal and Overweight Groups (**Table 6**).

**Table 6:** Post hoc multiple comparisons of muscular strength and endurance among normal, overweight, and obesity groups.

Indicator test	Group A	Group B	Mean group A	Mean group B	Difference (A-B)	P value
	Normal	Overweight	23.31	27.36	-4.04	0.065
Grip Strength, Left (kg)	Overweight	Obesity	27.36	25.79	1.57	0.471
	Obesity	Normal	25.79	23.31	2.47	0.257
	Normal	Overweight	24.66	28.92	-4.27	0.031*
Grip Strength, Right (kg)	Overweight	Obesity	28.92	28.76	0.17	0.933
	Obesity	Normal	28.76	24.66	4.11	0.038*
	Normal	Overweight	119.45	123.25	-3.80	0.679
Standing Long Jump (cm)	Overweight	Obesity	123.25	107.38	15.88	0.085
	Obesity	Normal	107.38	119.45	-12.08	0.190
	Normal	Overweight	9.83	11.78	-1.95	0.049*
Sit-Ups (30 seconds, repetitions)	Overweight	Obesity	11.78	8.00	3.78	0.001**
	Obesity	Normal	8.00	9.83	-1.83	0.065
	Normal	Overweight	14.65	15.35	-0.70	0.385
30-Second Chair Stand Test (repetitions)	Overweight	Obesity	15.35	14.43	0.93	0.251
	Obesity	Normal	14.43	14.65	-0.23	0.780

*p<0.05 and **p<0.01.

### 3.3 Flexibility and coordination indicators

**Figure 4:**
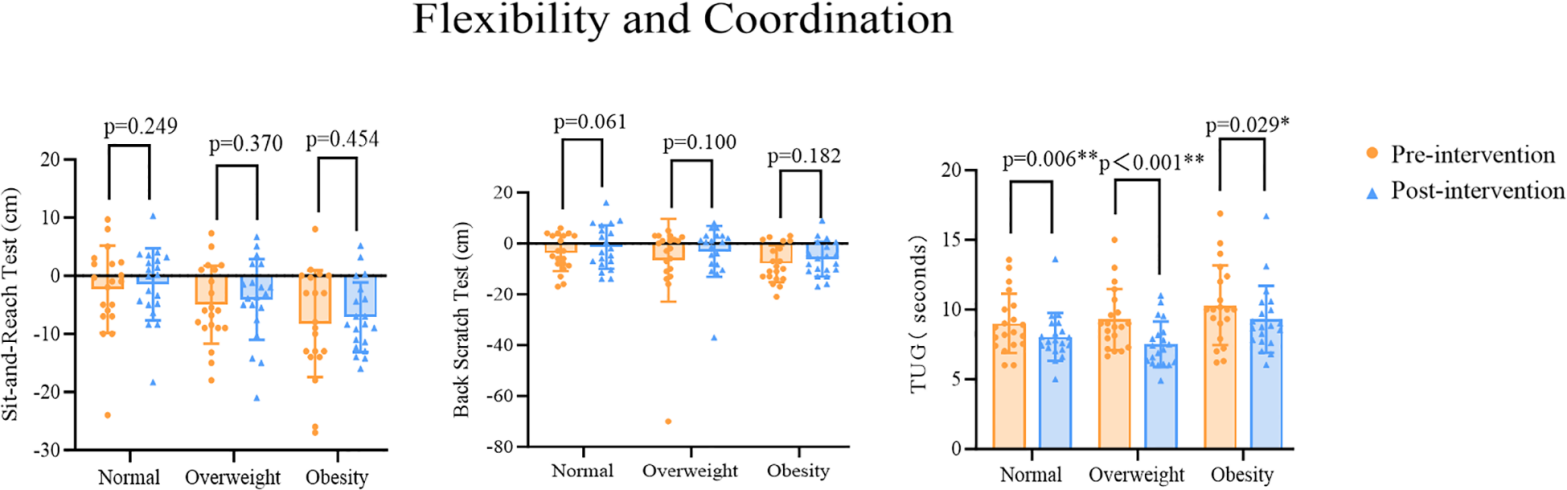
*p<0.05 indicates statistically significant differences, **p<0.01 indicates differences with greater statistical significance.

Paired sample t-tests were used to analyze changes in flexibility and coordination indicators across the three groups of participants. The results showed that compared with the pre-test performance within the same group, there were no significant differences in the post-test performance in the sit and reach test for the Normal (t=-1.189, p=0.249), Overweight (t=-0.918, p=0.370), and Obesity (t=-0.765, p=0.454) Groups. For the back scratch test, there were no significant differences in the post-test performances for the Normal (t=-1.992, p=0.061), Overweight (t=-1.728, p=0.100), or Obesity (t=-1.386, p=0.182) Groups compared with the pre-test performances. By contrast, for the TUG test, significant differences were found in the post-test performances for the Normal (t=3.071, p=0.006), Overweight (t=3.955, p=0.001), and Obesity (t=2.369, p=0.029) Groups (**Figure 4**).

MANOVA results showed that there were significant differences in some flexibility and coordination indicators between different BMI groups when comparing pre- and post-test performances. Further *post-hoc* comparisons (LSD) revealed the following results. For the sit and reach test, there were no significant differences in the changed performances between the Normal and Overweight Groups or between the Overweight and Obesity Groups (Normal vs Overweight: p=0.101; Overweight vs Obesity: p=0.052). However, there was a significant difference in the changed performances between the Normal and Obesity Groups (Normal vs Obesity: p=0.001). The Obesity Group had lower performances on the sit and reach test than the Overweight Group, and the Overweight Group had lower performances on the sit and reach test than the Normal Group, with the Obesity Group’s performance on the sit and reach test significantly lower than the Normal Group’s. For the back scratch test, there was a significant difference in the changed performances between the Normal and Obesity Groups (Normal vs Obesity: p=0.047); however, there were no significant differences in the changed performances between the Normal and Overweight Groups or between the Overweight and Obesity Groups (Normal vs Overweight: p=0.315; Overweight vs Obesity: p=0.321). The Obesity Group had lower pre- and post-test performances on the back scratch test than the Overweight Group, and the Overweight Group had lower performances on the back scratch test than the Normal Group, with the Obesity Group’s performances on the back scratch test significantly lower than the Normal Group’s. For the TUG test, there were significant differences in the changed performances between the Normal and Obesity Groups as well as between the Overweight and Obesity Groups (Normal vs Obesity: p=0.010; Overweight vs Obesity: p=0.005). By contrast, there was no significant difference in the changed performances between the Normal and Overweight Groups (Normal vs Overweight: p=0.794). The Obesity Group had the longest time, followed by the Normal Group and the Overweight Group. The Obesity Group’s performance on the TUG test was significantly higher than both the Normal and Overweight Groups (**Table 7**).

**Table 7:** Post hoc multiple comparisons of flexibility and coordination among normal, overweight, and obesity groups.

Indicator test	Group A	Group B	Mean group A	Mean group B	Difference (A-B)	P value
	Normal	Overweight	-1.90	-4.55	2.65	0.101
Sit and Reach Test (cm)	Overweight	Obesity	-4.55	-7.70	3.15	0.052
	Obesity	Normal	-7.70	-1.90	-5.80	0.001**
	Normal	Overweight	-2.60	-4.84	2.24	0.315
Back Scratch Test (cm)	Overweight	Obesity	-4.84	-7.05	2.21	0.321
	Obesity	Normal	-7.05	-2.60	-4.45	0.047*
	Normal	Overweight	8.53	8.40	0.13	0.794
TUG Test (seconds)	Overweight	Obesity	8.40	9.81	-1.41	0.005**
	Obesity	Normal	9.81	8.53	1.28	0.010*

*p<0.05, **p<0.01.

## 4 Discussion

This study systematically explored the effects of a 12-week badminton intervention on the health and fitness of adults with mild to moderate ID across different BMI levels.

### 4.1 Comparison of aerobic capacity indicators before and after badminton intervention

The results of this study showed that our badminton intervention program significantly improved aerobic capacity in adults with mild to moderate ID, with the Overweight Group showing greater observed improvements. This finding is consistent with those in the study by Chen et al. (2021) (11), in which the characteristics of badminton (such as rapid movement, hand eye coordination, quick swinging, short bursts of explosive force, and constantly alternating intervals times.) promoted health improvements best in overweight and obesity populations (31). In badminton, when a player completes a rally, they need to move quickly between the front and back courts and adjust their position for the next shot. This type of aerobic exercise enhances cardiovascular function (such as lowering HR, increasing lung capacity, and improving the number of 2-minute step repetitions) and aerobic metabolism (32, 33), which indirectly reduces the cardiovascular health risks associated with obesity.

Among the three BMI groups, greater improvement was observed for resting HR in the Overweight Group compared with the Obesity Group. This could be due to the more moderate weight of the Overweight Group allowing for them to maintain aerobic metabolism within the HR zone during the training process, thus improving cardiopulmonary endurance (34). The multidirectional movement of badminton not only promotes oxygen intake but also optimizes the diastolic and systolic functions of the heart. In contrast, adults in the Obesity Group, with a higher body fat percentage, are more likely to reach the anaerobic threshold during the same intensity of exercise, leading to lactic acid accumulation, which may partly inhibit the cardiovascular adaptation effects (35). The multidirectional movements in badminton (such as running to both front and back courts) require higher cardiopulmonary endurance. Although all three groups showed improvements in the 2-minute step test and in vital lung capacity, no significant differences were found between the groups, suggesting that badminton has a general positive effect on aerobic capacity. This may be related to the appropriate intensity of the exercise and the generally low baseline cardiopulmonary function of individuals with ID (36).

### 4.2 Comparison of muscle strength and endurance indicators before and after badminton intervention

The results of this study found that our badminton intervention program significantly improved muscle strength and endurance in adults with mild to moderate ID, with the Normal Group showing greater improvement. The continuous use of stepping, jumping, hitting, and adjusting movement in different directions (left-right, front-back, and up-down) in badminton requires the collaboration of upper and lower limb strength and core stability, which promotes muscle strength and endurance development. This is similar to some of the findings of Garam et al. (2018) (37). For example, the significant improvement in right-hand grip strength (83% of the participants in our study were right-hand dominant) may be associated with the frequent forehand and backhand shots in badminton, which involve continuous engagement of the forearm muscles and grip strength. This not only strengthens the wrist and forearm but also improves the endurance of grip strength.

Among the three BMI groups, the Normal Group showed greater improvements in right hand grip strength compared with the Overweight and Obesity groups. This result may be related to individuals with normal BMI having lower body fat percentages and better muscle mass. Previous research has shown that individuals with normal BMI usually have higher basal metabolic rates and better muscle activation efficiency (38, 39), which enables them to absorb exercise stimuli more efficiently during activities such as badminton racket swings and footwork training, thereby promoting rapid improvements in strength and balance. This is similar to the findings of Alana et al. (2024) (40). Furthermore, lower body weight leads to less inertia interference during lower limb movements, resulting in further optimization of dynamic coordination performance.

### 4.3 Comparison of flexibility and coordination indicators before and after badminton intervention

The results of this study indicated that our badminton intervention program significantly improved coordination in adults with mild to moderate ID, with greater improvements observed in the Normal Group. The improvement in the TUG test performances also suggested a synergistic enhancement of lower limb strength, balance, and coordination following the intervention (31). The requirement for spatial judgment and movement coordination during badminton (41) may further enhance muscle control ability, leading to significant improvements in coordination indicators, such as the TUG test. This finding is consistent with previous studies that suggest that badminton training has significant benefits for overall health improvement (12). In contrast to coordination, flexibility did not improve with the intervention. Improved flexibility requires regular and sufficient stretching exercises. Individuals with ID may have deficiencies in motivation and compliance, such as being easily distracted or lacking initiative in sustained participation, as well as abnormal muscle tone, limited joint mobility, etc. These restrictions can lead to insufficient intensity and frequency of stretching exercises during training, thereby limiting the improvement in flexibility (42). This finding also suggests that BMI may reflect underlying health conditions that are not under control.

Although the Obesity Group also showed significant improvements in fitness, their progress as assessed with the coordination indicator, the TUG test, was less pronounced compared with the Normal and Overweight Groups. Malek et al. (2024) found that individuals with obesity adopt compensatory movement patterns, such as shorter stride lengths and gait adjustments, to maintain movement stability (43), which partly inhibits further improvements in coordination. A higher body fat percentage can increase inertia load, thereby putting more strain on the neuromuscular control system. However, the multidirectional movements and balance adjustments in badminton may still positively influence their balance, leading to an overall positive change in coordination indicators.

Our findings highlight the importance of designing personalized exercise interventions for individuals in different BMI groups. Exercise programs should be tailored to the characteristics of each BMI group to effectively enhance exercise efficiency and maximize health benefits.

## 5 Limitations

This study has several limitations: While the 12-week intervention period allowed for short-term improvements in fitness, the sustainability of the effects could not be assessed. The behavior of individuals with ID is highly influenced by environmental support, and without follow-up interventions, fitness improvements may experience a rebound effect. Future studies could extend the intervention period (e.g., 6 months to 1 year) and add a follow-up phase to assess long-term benefits. Additionally, factors such as diet intake, sleep quality, medication, and daily activity levels that may affect fitness were not controlled in our study. Thus, for example, changes in the dietary structure of individuals in the Overweight and Obesity Groups during the intervention period may have impacted the results. In addition, this study did not have a non-intervention control group for comparison to isolate the specific effects of badminton.

## 6 Conclusion

This study demonstrated that badminton exercise has a positive impact on the health of adults with ID, including in aerobic capacity, muscle strength, endurance, flexibility, and coordination. The results showed that there were differences in the effectiveness of fitness improvements among different BMI groups. The Normal BMI Group showed greater improvements in grip strength (specifically right-hand grip strength) and the ability to perform the Tug test, while the Overweight Group showed greater improvement in the TUG test and greater improvement in HR than the Obesity Group. These findings suggest that individuals with BMI values lower than the obesity category may experience more substantial improvements across various aspects of fitness. Therefore, although badminton is an appropriate exercise intervention for individuals with ID, BMI and related factors should be taken into consideration when designing personalized exercise programs to optimize training effects.

## Data Availability

The original contributions presented in the study are included in the article/supplementary material. Further inquiries can be directed to the corresponding author.
